# Differential Effects of Social and Digital Feedback Interventions in the Figural Analogies Learning Test (FALT)

**DOI:** 10.3390/jintelligence14070134

**Published:** 2026-07-02

**Authors:** Adrian Büchli, Jens F. Beckmann, Stefan J. Troche

**Affiliations:** 1 Division of Personality Psychology, Differential Psychology and Diagnostics, University of Bern, 3012 Bern, Switzerland; stefan.troche@unibe.ch; 2 School of Education, Psychology and Education Cluster, Durham University, Durham DH1 3LE, UK; j.beckmann@durham.ac.uk

**Keywords:** dynamic testing, academic achievement, reasoning ability, learning test, figural analogies, digitalization

## Abstract

Dynamic testing (DT) aims to overcome a limitation of static assessment by providing learning opportunities as part of the test procedure so as to allow an individual’s latent potential to be evaluated rather than relying on a mere snapshot of their performance. Despite strong theoretical support, DT remains underutilized in research and applied settings. Thus, this study analyzes whether the Figural Analogies Learning Test (FALT), a newly developed short-term learning test (a DT procedure), is suitable for the assessment of latent potential in school-aged children. Mode of delivery for learning opportunities (i.e., feedback) was experimentally manipulated to investigate whether a digital or social modality results in differential effects on learning gains. A further aim was to establish whether—by capturing test takers’ latent potential—the FALT has incremental predictive utility in relation to academic achievement. The study randomly assigned 279 Swiss schoolchildren to one of three conditions: digital FALT, social FALT, or a static analogies task, all based on the same item set. Intervention effectiveness was evaluated using a static retest two weeks later. In both FALT conditions (digital and social), children achieved higher scores at the repeated assessment than in the static control condition. Moreover, socially mediated feedback produced significantly larger score gains than digitalized feedback. The three conditions did not differ in their ability to predict academic achievement and, thus, showed no incremental predictive utility for the FALT. However, an explorative exclusion of children with high grade averages indicated an advantage for the digital FALT relative to its static counterpart. The results provide tentative evidence that while both digitally and socially mediated feedback enhance learning performance, the latter is more effective. We discuss the absence of incremental predictive utility, specifically in the context of self-regulated learning, as well as implications for the design and diagnostic use of learning tests in educational contexts.

## 1. Introduction

Within educational psychology, measures of reasoning ability have been shown to be strongly associated with school grades, or academic achievement in general (Deary et al., 2007; Hofer et al., 2012; O’Connell, 2018; Peng et al., 2019; Roth et al., 2015; Zaboski et al., 2018). Beyond academic outcomes, school grades and reasoning ability are also linked to (economic) income and mental health in emerging adulthood (Borghans et al., 2008; Gottfredson & Deary, 2004; Starr et al., 2024). Consequently, insights into reasoning ability of children and adolescents are essential for a better understanding of their developmental trajectories.

In most reasoning tests, items are presented in a fixed order without feedback, so they are called static tests. Although the success of such measures in psychological practice has been clearly demonstrated, this static nature has also been criticized for limiting their capacity to capture intraindividual variability in cognitive functioning (Tzuriel, 2021). Theoretically rooted in Vygotsky’s zone of proximal development (Elliott et al., 2018; Guthke, 1982; Vygotsky, 1978), *dynamic testing* (DT)^1^ addresses this limitation by shifting the focus from an ability status to a test taker’s latent potential or learning ability (Guthke, 1982). *Learning ability* in this context is understood as a constituent aspect of reasoning ability, providing a diagnostically useful property that varies both between and within individuals (J. F. Beckmann, 2001). DT operationalizes intraindividual variability (i.e., learning ability) by embedding systematic learning opportunities (e.g., feedback, retries, and training) into the assessment process, thereby evoking and capturing performance change that remains inaccessible in static testing (J. F. Beckmann, 2014; Guthke, 1992; Guthke et al., 2003; Tzuriel, 2021). Importantly, DT does not aim to enhance reasoning ability or to assess a novel construct, but rather to achieve a more comprehensive representation of reasoning ability as it had originally been conceptualized (J. F. Beckmann, 2001, 2006). DT procedures have been shown to capture variance in ability beyond what is explained through static assessments, including, but not limited to, the prediction of school grades (Guthke & Stein, 1996) and test performance one year later (J. F. Beckmann, 2006). Moreover, static assessments may underestimate the abilities of children from disadvantaged backgrounds due to restricted prior learning opportunities, a limitation that DT is designed to mitigate (Caffrey et al., 2008; Elliott et al., 2018; Guthke et al., 1997; Guthke & Stein, 1996; Hessels, 2000; Resing et al., 2000). Nevertheless, these dynamic tests still play a minor role in psychological assessments, which is surprising given their closer alignment with the dynamic and adaptive nature of human behavior (J. F. Beckmann, 2014; Elliott et al., 2018; Hill, 2015). Comprehensive reviews of DT methodologies and approaches have been provided by Elliott (2003), Grigorenko and Sternberg (1998), Guthke and Stein (1996), Lidz and Elliott (2000), or Tzuriel (2021).

One of DT’s central limitations is its time-intensive nature, especially when the so-called *pretest–training–posttest* format is used. With this format, a baseline is established through an initial static assessment, followed by standardized training, and concluded by a posttest to measure score gains. The change in performance from pre- to post-test serves as an indicator of learning ability. In *short-term learning tests*, the training (and thus learning) phase is built directly into the testing procedure, thereby eliminating the necessity of a repeated measurement (Guthke, 1982). Typically, short-term learning tests involve an adaptive structure where incorrect responses trigger immediate assistance, redirecting test takers toward learning items that provide specific feedback and scaffolding. Learning ability (*intellectual change potential*) can then be operationalized as the number of errors and the extent of assistance required for subsequent success (J. F. Beckmann, 2001; Guthke & Stein, 1996). Two test takers achieving similar scores in a static assessment could still differ in their change potential: one may master a concept after a single prompt, while the other may require extensive support. Examples for such short-term learning tests can be found in the *Adaptive Computer-Based Intelligence-Learning Test Battery* (ACIL; Guthke et al., 1995). In response to the call for more systematic and controlled research on DT (Elliott, 2003; Elliott et al., 2018), the present research aims to revitalize the field, outline practical considerations for providing feedback so that it is effective, and offer new insights into how feedback functions in short-term learning tests.

Given that short-term learning tests (and DT procedures more generally) depend critically on effective intervention through feedback, the structure, standardization, and mode of feedback delivery require careful consideration. Feedback has been shown to be more effective when it provides specific information and elaborates on the underlying cognitive processes, thereby enabling learners to better understand the rationale for the incorrectness of their initial response (Wisniewski et al., 2020). Importantly, feedback effectiveness also depends on its alignment with the learner’s current position in the learning process, rather than being applied uniformly across all phases (Hattie & Timperley, 2007). In line with these requirements and drawing on feedback intervention theory (Kluger & DeNisi, 1996), N. Beckmann (2004) identified core components of effective feedback for DT frameworks. Specifically, feedback in a dynamic testing process should (1) correct erroneous problem-solving strategies, (2) go beyond simple correct vs. incorrect information, (3) include the correct item solution, (4) be delivered in a computer-based rather than examiner-administered format, (5) contain neither praise nor reprimand, (6) allow intraindividual performance comparisons by indicating changes over time, and (7) avoid promoting interindividual, norm-referenced comparisons. Together, these criteria provide tangible guidelines for designing effective feedback within a DT framework.

Due to advantages in standardization, scalability, and cost efficiency, DT implementations have increasingly relied on computerized and digital formats (Elliott et al., 2018; McNeil, 2018; Navarro & Mourgues-Codern, 2018; Poehner et al., 2015; Resing et al., 2015; J. Zhang & Lu, 2019; R.-C. Zhang et al., 2017). This aligns with N. Beckmann’s (2004) recommendation to use digitalized feedback. However, the empirical evidence supporting the superiority of digital feedback delivery may have evolved over the past two decades, particularly considering potential novelty effects associated with technology use in the late 1990s and early 2000s. For example, Wisniewski et al.’s (2020) meta-analysis did not find any meaningful differences in mode of delivery (oral, written, and computerized) on feedback effectiveness. Similarly, no differences were detected in the effectiveness of human and digital tutoring systems (Ma et al., 2014; VanLehn, 2011). Importantly, a potential conflict with the foundational developmental theories of DT may arise from a digitalized format. Both the zone of proximal development (Vygotsky, 1978) and the mediated learning experience (Feuerstein, 1990) conceptualize the driving force of cognitive growth as an interaction with a social partner. Whether described as “problem solving under adult guidance” (Vygotsky, 1978, p. 86) or “training given … by an experienced adult” (Feuerstein, 1969, p. 6), these theories posit that human learning is fundamentally rooted in social interaction. As Tomasello (2019) has put it, humans, especially young individuals, are “wired” to learn through social interactions, a quality that may be lost in purely digitalized environments. While digital feedback has been shown to foster students’ persistence, human-mediated feedback appears particularly effective at enhancing emotional engagement and the perceived credibility of the information (Heindl et al., 2025). This social advantage is further suggested by performance gains in tasks of greater difficulty, where the attribution of feedback to a human source, rather than a computer, has been associated with better outcomes (Spatola et al., 2021). Indeed, the fact that many digital feedback models are designed to emulate social interaction (e.g., Mayer & DaPra, 2012) underscores the theoretical importance of the social partner in the learning process. While, to the best of our knowledge, no studies have experimentally compared digital and human feedback effectiveness in a DT procedure, these results suggest that socially mediated feedback may be better suited to foster learning and thus improve performance. Consequently, for DT, we want to revisit the assumed superiority of a digitalized modality to a socially mediated one. Given the significant advantages of digital testing, such as standardization, cost-efficiency, and ease of application and scoring (requiring significantly less specialized training for test administrators), digitalized options remain the preferred long-term solution for dynamic testing.

The present study, therefore, investigates whether the mode of feedback delivery influences its effectiveness in a dynamic test for reasoning ability. For this purpose, a new short-term learning test, the *Figural Analogies Learning Test* (FALT), was constructed, which parallels the *Adaptive Figure Series Learning Test* (ADAFI; J. F. Beckmann, 2001; Guthke et al., 1995) of the ACIL. As in the ADAFI (J. F. Beckmann, 2001; Guthke et al., 1995), the FALT employs an adaptive testing procedure: after an incorrect response, test takers will be confronted with items of similar complexity. Within these so-called learning items, feedback is provided in a graduated manner, increasing in elaboration following repeated errors (N. Beckmann, 2004; Hattie & Timperley, 2007; Wisniewski et al., 2020). The feedback is structured into four hierarchical levels of increasing specificity. It transitions from a simple prompt to retry at Level 1 to error-specific instructional animations at Level 2. This is followed by a comprehensive visualization at Level 3, and finally, the presentation of the correct solution for reflection at Level 4.

In the study reported here, a sample of fifth- and sixth-grade children received this feedback in a computer-based format (digital FALT condition). Another sample received structurally equivalent feedback administered by a trained instructor (social FALT condition). To replicate previous studies and to examine whether the FALT provides information beyond a static assessment (J. F. Beckmann, 2006; Guthke & Stein, 1996), a third sample completed a static test using the same item pool but without feedback and training items (static control condition). To evaluate feedback intervention effectiveness and learning effects, test takers in all three conditions completed the static test after a two-week interval. Based on the theoretical considerations of dynamic testing and socially/digitally mediated learning, we tested the following hypotheses:

**Hypothesis 1 (intervention** **effectiveness).** *(a) In both FALT conditions (digital and social), test takers will achieve higher scores at a repeated static assessment than test takers in the static control condition, demonstrating the general effectiveness of the feedback intervention. (b) We anticipate that socially mediated feedback is more effective than digitalized feedback for performance, evidenced by higher scores for the social FALT condition at the repeated measurement compared to the digital FALT condition*.

**Hypothesis 2 (incremental predictive** **utility).** *(a) As in previous studies (J. F. Beckmann, 2006; Guthke & Stein, 1996), scores in the social as well as the digital FALT condition demonstrate stronger associations with school grades than scores from the static control condition. (b) If the FALT intervention promotes learning-related transfer effects, repeated-assessment scores in the FALT conditions should more closely reflect children’s academic achievement than scores in the static control condition. Accordingly, static test scores at the repeated assessment are expected to show stronger associations with school grades when the initial assessment was dynamic (digital or social) rather than static*.

## 2. Materials and Methods

### 2.1. Sample

The study was conducted with twenty-two classes from ten Swiss schools, with a two-week interval between the first (T1) and second (T2) test administrations. Of the 305 children who provided data at both measurement points, 13 were excluded due to entirely missing grade sheets, and another 13 were excluded due to more than one missing grade for the grade average (see Section 2.2.3). The final sample, therefore, consisted of 279 children in 5th and 6th grade. The mean number of test takers per class was 12.7 (*SD* = 3.9). For the total sample and the three subsamples, Table 1 shows the number of children, their self-reported gender, their mean age (and standard deviation), and how many children were in the fifth or sixth grades. The last column of Table 1 shows the mean number of days between the first and the second testing (and the corresponding standard deviation).

The prerequisite for testing children was the written informed consent of their parents. Children received a small bag of sweets for participating at both time points, and each participating class was awarded a diploma for classroom display and 100 CHF.

### 2.2. Measures

#### 2.2.1. Static Figural Analogies Test

**Figure 1:**
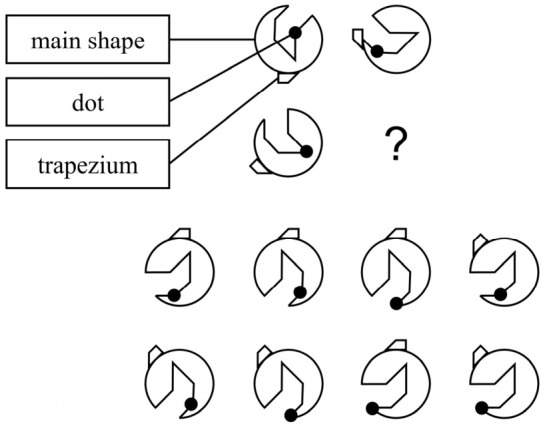
An exemplary item of the figural analogies containing three rules (main shape and trapezium rotation as well as dot movement), the solution is the 4th answer in the second row.

The figural analogy items were constructed using the IMak package (v2.1.0, Blum & Holling, 2018) in R (v4.4.1, R Core Team, 2024) and RStudio (v2024.9.0.375, Posit Team, 2024). A two-by-two matrix of figures was displayed for each item. The bottom-right cell contained a question mark, indicating that the cell was to be completed using one of the eight answer options displayed below (see Figure 1). Each individual figure in the matrix consisted of three elements, namely a main shape, a trapezium, and a dot. To solve these matrix problems, test takers had to identify the rules applied to the figures in the first row^2^ of the matrix and apply them to the second row. All items were presented in a two-part sequence. First, only the problem matrix was displayed, along with written instructions directing test takers to generate the solution mentally. Once ready, test takers could advance to the screen on which the matrix and answer options were presented (Figure 1) and submit their selected answer.

The set of possible *rules* that had to be identified in these matrix problems included (1) rotations of the trapezium around the main shape, (2) rotations of the main shape itself, and (3) edge movements of the dot on the interior of the main shape. For each of the three rules, the direction of movement and the amount of movement could be varied across items. Items could contain any combination of one to three of these rules. The test consisted of 16 items, presented in order of increasing complexity: first, three pairs of single-rule items (dot, trapezium, and main shape, respectively); followed by three pairs of two-rule combinations (dot/trapezium, trapezium/main shape, and dot/main shape, respectively); and a final four items incorporating all three rules simultaneously. Consistent with a static assessment format, test takers received no correctness indicators or feedback throughout the test.

Internal consistency for the Static Figural Analogies Test was evaluated using McDonald’s Omega total (ω) (McDonald, 1999). At T1 (static control condition only; *N* = 90), the scale demonstrated satisfactory internal consistency; ω = 0.78. At T2, using the full sample (*N* = 279), internal consistency remained satisfactory; ω = 0.85. Additionally, the test–retest reliability over a two-week interval within the static control condition was *r* = 0.71.

#### 2.2.2. Figural Analogies Learning Test

**Figure 2:**
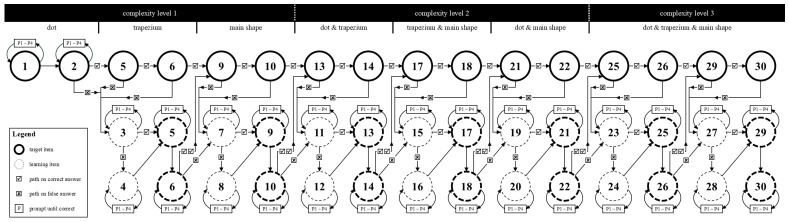
Layout and structure of the FALT with the sixteen target items in the first row (ordered by complexity) and the branches to the learning items after incorrect answers in the rows below. In Items 1 and 2, feedback was provided despite these being target items, to prevent children from beginning the test with errors and receiving no assistance on their initial attempts.

Within the FALT, test takers worked on the same 16 items as in the Static Figural Analogies Test and in the same order, referred to as *target items* (see upper row in Figure 2). Whenever test takers made an error on a target item (e.g., item 9), they did not directly advance to the next target item but were redirected to a *learning item* (in this case, item 7). Those learning items were parallel to the target items. For learning items, item isomorphs were created that contain the same psychometric characteristics (i.e., rules), referred to as *radicals*, but differ in their design features (i.e., starting point of each element), referred to as *incidentals* (Irvine, 2002). By using the same radicals and substituting incidentals, parallel test items were created that contained the same psychometric characteristics (Arendasy & Sommer, 2013; Blum & Holling, 2018). After an incorrect response was given to a learning item, feedback was then provided in a graduated manner, with increasing levels of information and elaboration contingent on repeated errors. Test takers could repeat a learning item up to four times until the correct solution was shown. Importantly, at every repetition of the same item, the answering options were pseudo-randomly rearranged to inhibit simple response-elimination strategies. After the initial learning item, progression depended on performance: test takers who responded without errors were returned to the target item at the same level of complexity (in this example, item 9), where it was presented as a learning item with graduated prompts as needed, whereas those who made errors were directed to a second learning item (item 8). If test takers solved both repetitions of the target items (items 9 and 10) correctly on their first attempt, they were directed towards the next target items in the next level of complexity (item 13), whereas errors within the repetitions of target items directed them towards the first learning item of the next level of complexity (item 11).

**Figure 3:**
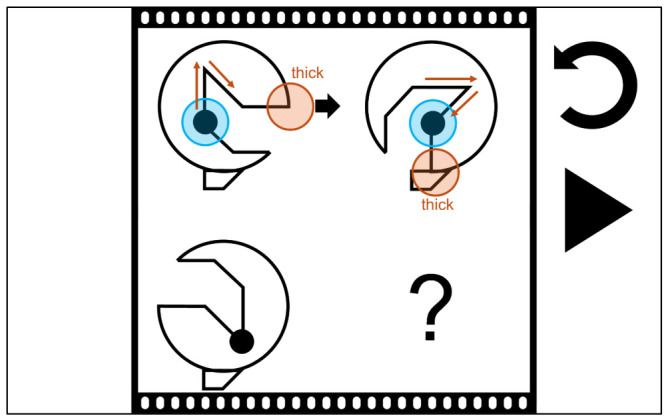
Screenshot of a tablet during Level 3 feedback visualizing the dot-rule in a dot-/main-shape-rule item. The term “thick” refers to one of the ends of the main shape (“side” of the opening). This terminology was used to determine the direction of the dot’s movement. The opposite end was referred to as the “thin” end. The video continued to illustrate the main-shape rule and subsequently applied both rules to the bottom figure, with the missing figure appearing on screen. Test takers could use the “reverse arrow” button to replay the previous segment of the instructional video and the play button to advance to the next segment.

The feedback provided within the learning items was divided into four distinct levels of specificity. After the first error within a learning item, test takers received Level 1 feedback, which informed them of the incorrect response and encouraged them to attempt the item again (*“review the item once more and try to find the solution”*). After a second error on the same item, Level 2 feedback was provided in the form of an instructional video tailored to the specific error type (e.g., if the dot was misplaced, only the dot rule was explained). This feedback followed a standardized structure: (1) directing the test takers’ attention to the erroneous dimension, (2) clarifying how the relevant rule should be applied, and (3) encouraging another attempt. Following a third error on the same item, Level 3 feedback was delivered. At this depth, feedback was no longer differentiated by error type^3^. Instead, an instructional video demonstrated, in sequential order, how all rules embedded in the item must be applied to reach the correct solution, which was accompanied by another prompt to retry the item (see Figure 3). After a fourth error, Level 4 feedback was presented, which provided the correct solution along with instructions to reflect on why this option was correct. Importantly, all instructional videos (Levels 2 and 3) displayed an image of a prototypical item (item isomorph; see previous paragraph). Thus, feedback was not provided directly on the specific item the test takers were working on. Furthermore, if an incorrect answer later in the FALT would have triggered a repetition of an instructional video (Levels 2 and 3), test takers were given the option to skip the video.

The feedback videos were created using Microsoft PowerPoint and an AI-generated voice (ElevenLabs, n.d.). Because Level 2 feedback required videos explicitly tailored to specific error types in each complexity level, a total of 26 distinct videos were developed. Including a detailed description of all videos in the main manuscript would exceed reasonable space constraints; therefore, a comprehensive description and breakdown of the wording are provided on the osf.

The operationalization of test takers’ intellectual change potential for the FALT was based on the approach adopted for the ADAFI (J. F. Beckmann, 2001; Guthke et al., 1995). Performance was quantified by the total number of steps taken to complete the test; a higher step count indicates lower ability and potential, as each repeated attempt due to an error increments the total (e.g., four attempts at item 7 contribute four steps to the final score). The minimum possible value is 16, while the maximum is 120. However, due to time constraints during test administration, not all test takers completed all target items, which could distort this measure. To account for this, a percentage was calculated representing the number of steps completed relative to the maximum possible steps up to the last item attempted, called the *step count*. In the sample used in this study, 95.24% reached the second level of complexity, while 35.98% reached the third level.

Assessing the reliability of a learning test is challenging, as the dynamic components are explicitly designed to evoke variability in the measured construct through intervention (Tzuriel, 2021), whereas classical test theory assumes stable true scores. Reliability was estimated using pairwise complete observations, such that item correlations were calculated using all available observations for each item pair. However, because some learning-item pairs were never jointly administered to participants, reliability estimation including all learning items resulted in structurally missing correlations. Within both FALT conditions, reliability was therefore estimated using only the target items, yielding an internal consistency of ω = 0.65. Exploratory analyses, including the learning items, yielded a higher estimate (ω = 0.77); however, these estimates relied on smoothing procedures and should therefore be interpreted cautiously. Correlation with the test score at T2 was *r* = 0.71.

#### 2.2.3. School Grades

School grades in Mathematics, German, French, English, NHS^4^, Sports, Arts and Crafts, and Textile Work were obtained from the annual report cards and matched to each test taker prior to anonymization. Since the study was conducted between September and December and the school year begins in mid-August, the annual report card from the previous school year was used, as grades from the current year would otherwise only contain one or two exams. Within the Swiss school system, a grade of 1 constitutes the lowest possible grade, while 6 is the highest. A grade of 4 or higher is considered a passing grade.

As one of the goals of the current study was to investigate correlations with academic achievement in general, a grade average was calculated from grades in the subjects Mathematics, German, and NHS^5^ (cf., Brunner et al., 2025). In the Swiss school system, students may be exempt from standard grading if they are assigned reduced learning goals (RLG) for a specific subject. We applied a partial inclusion criterion: students with an RLG in only one of the three subjects were retained, with their RLG being replaced by the average of their existing grades. This resulting average represents the same value as their grade sheet, where RLG grades are omitted for the calculation of an average. As explained in the sample description, students with two or more RLGs were excluded from this study, as we deemed a single grade to be an insufficient proxy for overall academic achievement.

### 2.3. Procedure

School classes were informed about the study one to two weeks prior to the start of data collection. Children received a flyer, an information letter, and a consent form to take home for their parents. Written informed consent was obtained from parents via the signed forms, which were subsequently returned to the study administration. Once all consent forms were collected, the total number of participating children in each class was randomly assigned to one of three experimental conditions, ensuring balanced distributions to all conditions within school classes.

All assessments were administered via PsychoPy (v2025.2.4, Peirce et al., 2019) on Samsung Galaxy S7 FE tablets (12.4-inch display; 2560 × 1600 resolution) within a separate room at the participating schools, and tablets were propped up on individual tables. At T1, all test takers watched a four-minute instructional video on their respective tablets explaining the task and potential rules. Those in the digital FALT and static control groups were assessed in small groups (*M* = 3, maximum of 5) and wore Sennheiser HD 350BT Bluetooth headphones to minimize distractions. In contrast, test takers in the social FALT condition were assessed individually, with a trained instructor seated to the child’s right. In the digital FALT, videos played automatically with AI-generated narration; in the social FALT, trained instructors provided equivalent live narration while the audio of the tablet was muted. Notably, while the static control group performed the Static Figural Analogies Test, both FALT conditions utilized the exact same software version to ensure that only the delivery of feedback differed. The session concluded with all test takers completing a demographic questionnaire.

At the second assessment (T2), conducted two weeks later, all test takers completed the Static Figural Analogies Test and two additional questionnaires not relevant to the current study^6^. This included a repetition of the four-minute instructional video. Here, all testing was done in small groups. Both T1 and T2 assessments were time-limited to 32 minutes, with timing commencing immediately after the instructional video and encompassing only the figural analogies items, not the questionnaires. This time constraint was implemented to ensure that the entire session (instructional video, test, and questionnaires) did not exceed 45 min, consistent with the standard lesson duration in Swiss schools.

### 2.4. Statistical Analysis

Data analyses were conducted using R (v4.4.1, R Core Team, 2024) and the following packages: rstatix (v0.7.2, Kassambara, 2023b), tidyverse (v2.0.0, Wickham et al., 2019), psych (v2.4.6.26, Revelle, 2024), and cocor (v1.1.4, Diedenhofen & Musch, 2015). Data visualization was done using patchwork (v1.3.2, Pedersen, 2025) and ggpubr (v0.6.0, Kassambara, 2023a). This study was pre-registered on osf, for further information see the transpareny note. First, descriptive statistics (means and standard deviations) for all grades used in the grade average (Mathematics, German, and NHS) and test scores for both time points are reported.

To ensure randomization led to similar academic competencies between conditions, potential differences were first examined via a series of one-way ANOVAs on individual school grades (Mathematics, German, and NHS) and the grade average. This methodology was not preregistered, as we expected randomization to naturally lead to comparable ability across conditions (Senn, 1994).

To evaluate Hypotheses 1a and 1b, a one-way ANOVA followed by pairwise one-tailed *t*-tests was conducted on T2 scores to compare the learning effects across all three conditions. To test Hypotheses 2a and 2b, Pearson correlations (*r*) were calculated between grade average and T1 and T2 scores, respectively.

While the preregistration specified a directional comparison between two independent correlation coefficients, we did not explicitly name the statistical test; the inferential procedure follows directly from this hypothesis and is specified here for clarity. To formally compare the magnitude of correlations between conditions, we employed the cocor R package (v1.1.4, Diedenhofen & Musch, 2015) and the function cocor.indep.groups to compare correlation coefficients between independent groups, based on Fisher’s *r* to *Z* transformation (Fisher, 1925). Importantly, for direct comparisons of correlation coefficients, absolute values were used, as the direction of the correlations in the FALT conditions (digital and social) was opposite to that in the static control condition. Within the FALT, fewer steps in the structural path indicate higher ability, whereas in the Static Figural Analogies Test, higher ability is reflected by a greater number of correct items.

Prior to conducting any ANOVAs, the assumption of homogeneity of variance was evaluated using Levene’s test (Levene, 1960). In instances where this assumption was violated, Welch-corrected tests were employed, and adjusted degrees of freedom were reported accordingly (Welch, 1947). To quantify the magnitude of the observed effects, effect sizes are reported alongside *p*-values: generalized eta-squared ($\eta_{G}^{2}$) is provided for ANOVAs, and Cohen’s *d* is reported for comparisons using pairwise, one-tailed *t*-tests. Effect sizes were interpreted according to the benchmarks proposed by Cohen (1988): for $\eta_{G}^{2}$, values of 0.01, 0.06, and 0.14 represented small, medium, and large effects, respectively; for Cohen’s *d*, the corresponding thresholds were 0.2, 0.5, and 0.8.

## 3. Results

For the descriptive statistics, Table 2 presents the means and standard deviations for individual subject grades (Mathematics, German, and NHS), the overall grade average, and the primary test metrics. Specifically, these include raw scores for the static control condition and step counts for the FALT conditions at the initial assessment (T1) and raw scores for all conditions at the repeated assessment (T2).

To ensure academic competencies were comparable across conditions, we conducted a series of one-way ANOVAs on individual school grades (Mathematics, German, and NHS) as well as the grade average. Levene’s tests indicated that the assumption of homogeneity of variance was met for all analyses (all *F*_2, 276_ < 2.17, all *p*s > .12). One-way ANOVAs revealed no significant differences between the digital, social, and static conditions for any individual subject or the grade average (all *F*_2, 276_ < 0.79, all *p*s > .45). These results suggest that the three conditions entered the study with comparable academic ability levels.

Hypotheses 1a and 1b focused on the intervention effectiveness. They stated that (a) the FALT conditions elicited greater learning effects than the static control condition (as indicated by higher T2 scores) and (b) that this advantage was more pronounced in the social than the digital condition. To investigate these hypotheses, a one-way ANOVA was conducted on T2 scores (see Table 2). Levene’s test confirmed that the assumption of homogeneity of variance was met (*F*_2, 276_ = 0.613, *p* = .543). Performances in the static test at T2 differed substantially between conditions (*F*_2, 276_ = 15.869, *p* < .001, $\eta_{G}^{2}$ = 0.103). For the pairwise one-tailed *t*-tests, *p*-values were adjusted using the Bonferroni–Holm procedure to control the familywise error rate (Holm, 1979). The digital FALT condition yielded significantly higher scores than the static control condition (*p_adj_* = .006, *d* = 0.36). Furthermore, the social FALT condition demonstrated the highest performance, significantly surpassing both the static control condition (*p_adj_* < .001, *d* = 0.84) and the digital FALT condition (*p_adj_* = .002, *d* = 0.50).

Hypotheses 2a and 2b focused on the incremental predictive utility. They stated that performance in the digital and social FALT conditions would show stronger correlations with school grades than performance in the static control condition, both at (a) T1 and at (b) T2. To examine these hypotheses, Pearson correlations (*r*) were computed to assess the relationship between the grade average and test scores at T1 and at T2. The correlation coefficients are presented in Table 3. Results for separate grades (Mathematics, German, and NHS) are reported in Appendix A. However, contrary to our expectations, the correlations in the FALT conditions were descriptively lower than in the static control condition. Consequently, all one-sided comparisons were non-significant (all *Z* < −0.09, *p*s > .54). Thus, the null hypothesis was retained.

**Figure 4:**
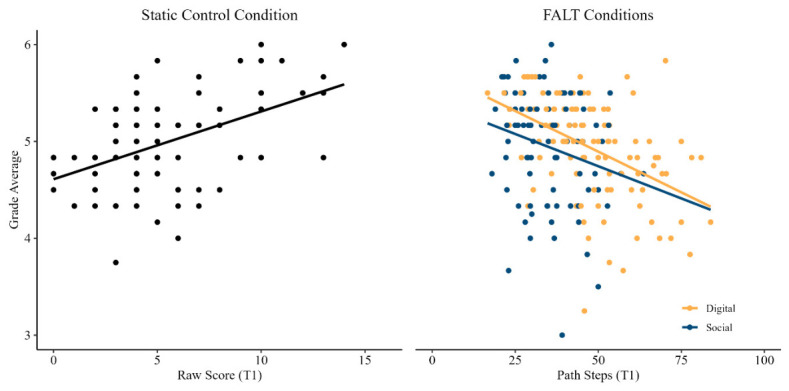
Scatterplot of grade averages and scores in the static control condition and the digital and social FALT conditions.

An exploratory visual inspection of the relationship between the grade average and scores at T1 suggested that a subset of high-performing students may have disproportionately influenced the correlations in the static control condition (cf., Figure 4). Thus, we explored whether students with high grade averages substantially influenced correlation coefficients at both time points. Table 4 reports the correlations of test scores and the grade average after students who exceeded one of three cutoffs were gradually excluded (>5.75; >5.5; >5.25). The explorations confirmed that correlations in the static control condition were to some extent driven by students with high grade averages, as the association between test scores and grade average weakened with increasingly restrictive cutoffs. In the digital FALT condition, this pattern was not observed. Although this provides insight into a potential limitation of the static control condition (and an advantage of the digital FALT), we refrained from formally comparing these adjusted correlations, as the artificial reduction in variance could bias statistical inference.

## 4. Discussion

The present study investigated whether the FALT, a learning test modeled on the principles of the ADAFI (J. F. Beckmann, 2001; Guthke et al., 1995), provided a comprehensive assessment of learning ability and thus captured not only interindividual differences in static reasoning ability but also intraindividual variability therein (latent potential). To test if the FALT was an effective learning intervention, we examined whether scores from a repeated assessment were higher for the FALT compared to the static control conditions (Hypothesis 1a). Of particular interest was the question of whether modality of feedback (digitalized and automatically administered versus socialized and individually administered) differentially affected the intervention effectiveness (Hypothesis 1b). Furthermore, we examined whether the elicitation and measurement of latent potential resulted in a stronger association with academic achievement (Hypothesis 2a) and if this effect translated to a repeated measurement (Hypothesis 2b).

### 4.1. Intervention Effectiveness

Consistent with Hypothesis 1a, test takers who worked on the FALT at an initial assessment demonstrated significantly higher scores at the two-week follow-up than those in the static control condition. First, this finding indicates that the instructional scaffolding embedded in the FALT achieved its intended effect of eliciting intraindividual variability in reasoning ability, enabling test takers to become more versed in solving figural analogy items. Thus, the test procedure realized in the FALT can be considered an effective learning intervention. In line with Vygotsky’s (1978) zone of proximal development, the provision of guidance enabled children in both FALT versions to demonstrate cognitive competencies that exceeded their performance when working independently. Second, although a two-week interval is relatively brief in the context of retesting research, retest effects are typically found to be reasonably stable over time (Hausknecht et al., 2007; Scharfen et al., 2018). Accordingly, the performance gains observed in the FALT versions are consistent with a durable effect rather than a purely transient spike in performance. This interpretation is further supported by the “first exposure” characteristics of the sample: approximately two-thirds of the children in the FALT conditions did not reach the final level of complexity during the initial FALT assessment and thus encountered three-rule items for the first time in the second testing session. Their comparatively stronger performance despite this limited prior exposure suggests generalized transfer of problem-solving strategies rather than simple item-specific memorization.

Beyond these overall effects, the effectiveness of the feedback differed across modalities: gains were significantly larger in the social FALT condition than in the digital FALT condition (Hypothesis 1b), indicating a stronger impact of human-mediated scaffolding. While few studies directly compared digital and human-mediated feedback (Lechermeier & Fassnacht, 2018), existing research has primarily focused on organizational contexts or adult populations (e.g., Alder & Ambrose, 2005). In such an adult sample, Kluger and Adler (1993) found that human feedback, compared to computer-delivered feedback, decreased performance when restricted to simple accuracy information. However, our findings suggest that human-delivered feedback outperforms digital feedback when feedback is elaborative rather than merely evaluative. This aligns more closely with the study by Golke et al. (2015), which determined that digital feedback had no effect on performance in text comprehension, whereas person-mediated feedback significantly boosted performance. Crucially, Golke et al.’s (2015) study utilized a one-on-one setting, distinguishing their work from studies using remote meetings, modalities where person-mediated feedback has been found to hinder performance (Merrick & Fyfe, 2024). While Golke et al. (2015) attributed their findings to social facilitation (Guerin, 1986), they did not include a no-feedback condition with an experimenter present to isolate this effect. In contrast, because an experimenter was present and observing the tablets across all our conditions, we argue that a more active mechanism was at play. Rather than mere presence, the constant interaction in the social FALT condition likely functioned as a form of external regulation. This *co-regulation* (Hadwin et al., 2011) likely minimized individual differences in motivation and prevented disengagement, encouraging students to persist through challenging items and to actually attend to the feedback, and thus, to profit more from the intervention (Zimmerman & Schunk, 2001). Such mechanisms would not have been present in the digital FALT or the static control condition, as the experimenter was present but did not interact with students during the task and solely observed. We therefore assume that the observed advantage of the social FALT condition was likely influenced not only by the feedback modality itself but also by differences in the interpersonal testing setting.

### 4.2. Incremental Predictive Utility

Contrary to our expectations (see Hypothesis 2a), the FALT did not demonstrate incremental predictive utility of school grades when compared to a static task using the same items. We assumed that the FALT provides a more comprehensive representation of children’s latent potential, which in turn shows stronger associations with academic achievement than static test performance, as demonstrated in previous studies using the ADAFI (cf. J. F. Beckmann, 2006; Guthke & Stein, 1996). Here, it is vital to first differentiate between feedback modalities. For the digital FALT, which more closely mirrors the ADAFI (J. F. Beckmann, 2001; Guthke et al., 1995), correlations were almost identical to the static control condition. Although both the learning test and the static task showed similarly strong associations with academic performance, the FALT provided an additional dimension of insight: the extent to which a child benefited from feedback and thinking prompts. Furthermore, an explorative analysis revealed that only the digital FALT retained any meaningful associations with academic performance once high-achieving students were removed from the analyses. This pattern was also observed for the test performance at T2, where all worked under static conditions. For high-achieving children, this may suggest that the additional information gained from a dynamic procedure is marginal. For lower-achieving children, this finding supports the notion that learning tests are more effective in differentiating amongst low performers (J. F. Beckmann, 2006; Guthke & Stein, 1996). Given that lower-achieving children are more frequently the focus of intelligence assessments, DT appears particularly advantageous in this context. Specifically, dynamic formats can help counteract common barriers, such as initial difficulties in understanding instructions and low motivation. However, these findings of differential predictive utility of the FALT are tentative and require replication in a larger sample, particularly given the artificial restriction of variance.

Meanwhile, the social FALT condition presents a compelling, if initially contradictory, result. Although socially mediated feedback appeared to be most effective in improving performance, the scores from the social FALT were descriptively less closely associated with the grade average than those from the static and digital FALT assessments. This stands in contrast to our hypothesis of incremental predictive utility. J. F. Beckmann (2006, 2014) described specific scenarios where a learning test may relate less strongly to external criteria, or even be expected to do so, while still maintaining its internal validity. In static assessments, success is, to some extent, determined by previous learning experiences. For test takers from disadvantaged backgrounds, these scores may reflect a lack of opportunity rather than a lack of intellectual potential (Guthke & Stein, 1996; J. F. Beckmann, 2001, 2014). Thus, DT aims to level the playing field by providing learning opportunities within the assessment itself. It is possible that the social interaction provided such efficient support that it effectively reduced performance gaps between children, which remain present in their school grades. This could be interpreted as “good news”: the social FALT may be capturing a dimension of cognitive potential that is currently untapped or obscured by traditional academic grading. Furthermore, unlike the social condition, digital FALT and static control conditions more closely resembled a typical classroom experience, where students must independently manage their own persistence and focus. Consequently, digital FALT and static control conditions may have shown stronger associations with academic achievement because they capture not only reasoning performance, but also children’s ability to self-regulate. In the social FALT condition, however, this ability to self-regulate may have been scaffolded through co-regulation, and thus partially obscured (N. Beckmann, 2004; Hadwin et al., 2011).

Regarding Hypothesis 2b, we found no statistically significant differences between the conditions in their correlations between school grades and test scores in the static test at the repeated measurement (T2). Given that those differences did not manifest at the initial assessment, it is unsurprising that they also did not appear at the repeated assessment. Overall, the indices with school grades were satisfactory, as they were similar to correlations usually found for tests of reasoning ability and school grades (Hofer et al., 2012; Peng et al., 2019; Zaboski et al., 2018).

### 4.3. Further Considerations and Limitations

Another important practical consideration concerns the time investment associated with each assessment format. Both the static test and the digital FALT were administered in small groups of up to five children without requiring active experimenter intervention. In contrast, the socially mediated FALT necessitated time-intensive, one-on-one administration and extensive training of each experimenter. This points to a clear trade-off: although the socially mediated FALT was most effective in revealing children’s learning potential, it required disproportionately greater resources, whereas the digital FALT demonstrates that dynamic testing can be implemented in fully digital formats, albeit capturing only part of this potential.

While this study provides valuable insights into DT, several limitations warrant consideration. First, the sample size was relatively modest. Although adequate for detecting large intervention effects, the study may have been underpowered to identify more subtle differences in correlation coefficients across conditions. Because the observed pattern for the correlational analyses was opposite to the hypothesized direction, no formal post hoc power analyses were conducted for these comparisons. A further limitation concerns the relatively low internal consistency of the FALT. This may be expected in dynamic testing contexts, given the intentional violation of the measurement maxim of “local stochastic independency” and subsequently the introduction of construct heterogeneity. However, lower internal consistency may also have attenuated observed effect sizes and reduced statistical power, particularly for the correlational analyses related to H2a and H2b. Although exploratory analyses including learning items increased reliability estimates (ω = 0.77), these estimates should be interpreted with caution due to sparse overlap between some item pairs. Future studies with larger samples and more complete item overlap are needed to obtain more robust reliability estimates for the FALT.

Another limitation concerns the exclusive use of school grades as the criterion measure. The relationship between grades and reasoning ability is well established, given their use in identifying the need for intelligence assessment, determining students’ grade placement, and their strong associations with intelligence. However, grades are also influenced by teachers’ expectations, classroom behavior, and other non-cognitive factors, and vice versa (Duckworth et al., 2012). Finally, the study used concurrent academic achievement, and while school grades tend to be relatively stable (Guskey, 2011; Scherrer et al., 2025), we cannot make any assumptions about a prediction of future academic achievement.

## 5. Conclusions

In summary, the present study demonstrated that the FALT is effective in eliciting intraindividual variability in reasoning performance in response to feedback (i.e., learning). The socially mediated FALT revealed that elaborative feedback provided by a person was more effective than digitally administered feedback. However, this did not translate into incremental predictive utility for academic achievement for either of the FALT conditions. While the exploratory analyses revealed an advantage of the digital FALT over the static assessment in predicting school grades, these findings should be interpreted with caution due to the artificial restriction in variance. Although the socially mediated FALT represented the most effective learning intervention, it resulted in a numerically weaker prediction of academic achievement. Future research should seek to disentangle the regulatory mechanisms potentially responsible for this pattern by directly comparing one-on-one FALT sessions with measures of academic achievement that incorporate more comparable settings. Moreover, incorporating additional external criteria such as standardized achievement tests and longitudinal teacher ratings that are specifically geared towards the perception of potential will be essential for further evaluating the validity and generalizability of learning test scores across diverse student populations.

## Figures and Tables

**Table 1:** Summary and descriptive statistics of the sample analyzed. The columns named 5th and 6th refer to the number of children in each respective grade, while the interval column refers to the average time interval between T1 and T2 in days.

Condition	N	Male	Female	Other	5th	6th	Age (*SD*)	Interval (*SD*)
Static Control	90	47	42	1	43	47	10.97 (0.71)	13.79 (1.34)
Digital FALT	107	56	50	1	52	55	11.05 (0.72)	13.86 (1.56)
Social FALT	82	43	39	0	39	43	11.05 (0.77)	13.17 (2.10)
Total	279	146	131	2	134	145	11.02 (0.73)	13.63 (1.69)

**Table 2:** Means and standard deviations (*SD*s) of all grades and test scores used in the study are split along conditions.

Condition	Mathematics		German		NHS		Grade Average		T1		T2	
	*M*	*SD*	*M*	*SD*	*M*	*SD*	*M*	*SD*	*M*	*SD*	*M*	*SD*
Static Control	4.90	0.61	4.99	0.48	5.06	0.52	4.98	0.47	5.30	3.29	6.40	3.90
Digital FALT	4.92	0.64	4.93	0.53	4.96	0.59	4.94	0.52	47.57 ^a^	14.57	7.79	3.77
Social FALT	4.90	0.73	4.97	0.57	4.96	0.67	4.94	0.59	34.98 ^a^	14.57	9.74	4.06

^a^ Measure at T1 is the step count.

**Table 3:** Pearson correlations of T1 and T2 scores with the grade average under the three conditions.

Condition	*N*	Time Point 1	Time Point 2
Static Control	90	0.484 ***	0.511 ***
Digital FALT ^a^	107	−0.474 ***	0.432 ***
Social FALT ^a^	82	−0.228 *	0.296 **

^a^ Measure at T1 is the step count. Correlations of grade average and step count (social and digital condition) are negative at T1 because fewer errors throughout the session lead to a lower step count. *** *p* < .001, ** *p* < .01, * *p* < .05.

**Table 4:** Pearson correlations of scores at T1 and T2 and the grade average, calculated in three reduced samples with students at different grading ceilings removed, for each condition, respectively.

Condition	<5.75			<5.5			<5.25		
	N	T1	T2	N	T1	T2	N	T1	T2
Static Control	84	0.372 ***	0.463 ***	73	0.155	0.241 *	63	0.135	0.195
Digital FALT ^a^	106	−0.513 ***	0.463 ***	88	−0.438 ***	0.356 ***	73	−0.337 **	0.261 *
Social FALT ^a^	79	−0.222 *	0.279 ***	62	−0.184	0.190	53	−0.103	0.076

^a^ Measure at T1 is the step count. Correlations of school grades and step count (social and digital condition) are negative because fewer errors throughout the session lead to a lower step count. *** *p* < .001, ** *p* < .01, * *p* < .05.

## Data Availability

The original data and R code used and presented in the study are openly available on osf at https://doi.org/10.17605/OSF.IO/CF5T8 (accessed on 22 May 2026). This study’s hypothesis, design, and parts of its analysis were pre-registered on osf https://doi.org/10.17605/OSF.IO/XM7QR (accessed on 22 May 2026). Although preregistered, we refrained from performing any Rasch Model analyses within this publication due to the sample size. We strive to perform these analyses with a larger sample. Furthermore, we opted to use the terminology of predictive utility rather than predictive validity within this publication, as that terminology has proven to be more fitting for our study.

## Notes

1 DT is sometimes used interchangeably with Dynamic Assessment (DA), and while DT and DA attempt to measure the same psychological processes, DT is more focused on the psychometric measurement process itself, while DA is more focused on the following interventions (Elliott et al., 2018).

2 While items could be solved using either a horizontal (first row; left-right) or vertical (leftmost column; top-down) strategy, the introductory video focused solely on the horizontal left-to-right solution path.

3 At this depth, the error type was no longer differentiated because to reach it required three errors to have occurred, making it difficult to reliably determine which single dimension was responsible.

4 NHS stands for *Nature, Humans, Society*, which covers the four domains (1) Nature and Technology, (2) Economics, Work, and Household, (3) Spaces, Times, and Societies, and (4) Ethics, Religions, and Community (Deutschschweizer Erziehungsdirektoren-Konferenz, 2016).

5 Grades in English and French were not used for the grade average because of missing data, as these subjects are not always being taught in 4th grade, while Sport, Textile Work, and Arts and Crafts tend to be less associated with reasoning ability (Roth et al., 2015).

6 Need for Cognition Teens (NFC-Teens) (Preckel, 2016); Questionnaire on Achievement Motivation for Students in Grades 3 to 6—Revision (FLM 3-6 R) (Lohbeck & Petermann, 2019).

## Abbreviations

The following abbreviations are used in this manuscript:

DT	Dynamic Testing
FALT	Figural Analogies Learning Test
NHS	Nature, Humans, Society
ACIL	Adaptive Computer-Based Intelligence-Learning Test Battery
ADAFI	Adaptive Figure Series Learning Test

## Appendix A

In Table A1, the Pearson correlation coefficients are reported for test scores at both time points (T1 and T2) with each of the three grades used for the grade average. Furthermore, differences in correlation coefficients for both time points and grades in Mathematics, German, and NHS were calculated and reported. None of those differences reached statistical significance (all *F*s < 0.51, all *p*s > .31). Therefore, for specific grades, results did not differ from those obtained from the grade average.

**Table A1:** Pearson correlations between T1 and T2 scores of all conditions and all graded subjects. Correlations of school grades and step count (social and digital condition) are negative at T1 because fewer errors throughout the session lead to lower step counts.

Condition	Mathematics	German	NMG
Time point 1			
Static Control	0.483 ***	0.408 ***	0.392 **
Digital FALT	−0.538 ***	−0.409 ***	−0.457 ***
Social FALT	−0.218 *	−0.243 *	−0.239
Time point 2			
Static Control	0.556 ***	0.363 ***	0.406 ***
Digital FALT	0.446 ***	0.420 ***	0.272 **
Social FALT	0.367 ***	0.218 *	0.202

*** *p* < .001, ** *p* < .01, * *p* < .05.
